# Commentary on the 6th International Symposium of Animal Functional Genomics 

**DOI:** 10.1186/s12711-016-0276-z

**Published:** 2016-12-09

**Authors:** Paolo Ajmone-Marsan, Alessandra Stella

**Affiliations:** 1 Institute of Zootechnics, Università Cattolica del Sacro Cuore, Piacenza, Italy; 2 Nutrigenomics and Proteomics Research Center, Università Cattolica del Sacro Cuore, Piacenza, Italy; 3 National Research Council, Lodi, Italy; 4 Parco Tecnologico Padano, Lodi, Italy

On the year of Expo 2015, “Feeding the Planet”, prominent scientists from different countries and with a wide range of expertise convened at the Università Cattolica del Sacro Cuore in Piacenza to attend the 6th International Symposium on Animal Functional Genomics (http://www.isafg2015.it) and discuss the role of functional genomics and post-genomic perspectives in animal breeding. Topics included genome architecture and regulation, the role of microbes in health and production, and the best use of new technologies for improving livestock sustainability in developed and developing countries. One major achievement of the symposium was full integration of the separate sessions through a very interactive exchange among representatives of academia, biotech industries and breeding organizations.

Interestingly, strategies for the investigation of topics as different as detection of rare disease variants or animal breeding in developing countries progressively converged on the need to implement, improve and share theories and tools on functional prediction of genomic variance in complex traits.

Genomic technologies were concluded to be sufficiently mature to expand their application in the field to uses beyond genomic selection in cosmopolitan dairy cattle. Beef breeds and other species, such as pig and sheep, are expected to progressively increase the use of DNA information in breeding programs. The availability of precise phenotypic data is fundamental in this endeavour. Therefore, in developing countries, the success of genomic approaches strongly depends on the type of husbandry system and the level of organization of animal breeding activities (Kathiravan Periasami and Jose Fernando Garcia). Besides accurate phenotype recording, new methods are needed to account for the lesser availability of DNA and phenotypic data in small populations. Novel methods for across-breed evaluation, for example, may provide a suitable solution for local breeds. In such small populations, genomics may also inform programs for conservation of genetic diversity and facilitate the identification of major genes associated with specific negative or positive characteristics such as genetic defects or adaptation. Whole-genome sequencing (WGS) greatly facilitates this exercise. Paradigmatic examples are the quests for the identification of causal variants for transverse hemimelia in buffalo [1] and for the *SLICK* mutation that increases heat tolerance in the Caribbean Senepol and Carora cattle breeds (Tad Sonstegard). WGS also permits the reconstruction of species and breed origins, demography and admixture and the identification of genomic regions under positive and balancing selection, which carry relevant genes for production and adaptation [2].

Conversely, the identification and use of genes that control complex traits is still more elusive. Even in humans, where many thousands of samples have been sequenced or genotyped with millions of SNPs, genes identified for quantitative traits explain only a limited proportion of the trait variance and their cumulative effect is small (Nicole Soranzo). Alternatives have been discussed, such as tagging “intermediate phenotypes” that are components of complex traits, the concentration of specific compounds in milk that contribute to milk quality and yield (Ben Hayes) or immune repertoire profiling as component of disease resistance (Tim Smith).

Understanding how genome variants affect phenotypes of complex traits involves the analysis of gene regulation mechanisms. Thus, annotation of livestock genomes with sites that have important regulatory functions is essential. The FAANG (Functional Annotation of Animal Genomes, Alan Archibald) initiative is guiding this endeavour: hundreds of researchers worldwide cooperate in producing, according to shared standards, functional data on livestock species, based on the model set by the human ENCODE project.

A further step in the investigation of the genetics of livestock focuses on the epigenetic control of gene expression. MicroRNAs have been proven to influence livestock and human disease status by post-transcriptionally controlling gene expression (John Williams). However their mechanisms of recruitment, regulation, and action are still not completely understood [3]. The control of gene expression by DNA methylation appears extremely important during embryo development and a major cause of failure for embryos obtained in vitro [4].

Since many terabytes of data are produced by different—omic technologies every day, bioinformaticians around the worlds work together at connecting, integrating and interpreting this information. Computational and systems biology together with system genetics informatics are exploring efficient strategies; novel network analysis and visualization tools support data exploration and interpretation and, concurrently, enrich the large public databases. These new approaches are efficient in investigating traits important for humans as well as livestock, such as response to vaccination, obesity, fertility and feed efficiency [5, 6].

Bioinformatics is the fulcrum for connecting information from the genome variants to post-translational modification, comparatively across species and, more recently, integrating data on the microbiome, through meta-genomics.

The gut microflora is now considered to be an integral part of the animal body and to have an important role in animal and human health and in nutrient absorption, likely influencing feed efficiency and obesity (Lorenzo Morelli). Animal/microbe interaction is particularly important in ruminants. On the one hand, rumen microbioma permit animals to survive on a high fiber diet by fermenting cellulose. On the other hand, some species of micro-organisms, archebacteria in particular, produce polluting gases such as methane and carbon dioxide that contribute substantially to the so-called “greenhouse effect”. The interaction between the rumen microbioma and the genetics of animals is presently under investigation, to explore the opportunity of developing ruminants with low methane emission and high feed efficiency (John Wallace).

Advances in technologies drive advances in science and vice versa. Reliability, accuracy, throughput and affordability of genomic and post-genomic technologies are improving, but further development is needed, which will only be possible through close collaboration between biotech industries and the research community (Affimetrix, Illumina, Agilent, Recombinetics, Flowmetric).

## Major conclusions in terms of policy relevance

The final discussion focussed on the large gap in available knowledge and the challenges that this research sector has to face in the near future to speed up and precisely guide selection and to customize animal management to increase livestock efficiency, welfare and animal product quality while decreasing the environmental impact of livestock production.

The completeness and precision of genomic information is one of the major gaps. Reference genomes are still imprecisely assembled and their annotation is largely incomplete, particularly in terms of variants that have a regulatory role and the variation of which has been demonstrated to be at least as important as structural genomic variation for the control of complex traits in humans. Also, costs are still too high for whole-genome sequencing of large livestock populations, while large numbers are needed for sound statistical analyses.

Genomics realizes its full potential if combined with high-throughput phenotyping; however, phenotypic data are presently limited to a few traits, often controlled by multiple and complex biological mechanisms. The collection of new and more precise phenotypic data including “intermediate phenotypes” that are related to the animal’s biology would close a second important gap of knowledge and greatly increase the understanding of livestock biology. Among the new traits, gut microbiota, particularly in the rumen, is a major focus of study because the methane eructed by animals contributes to air greenhouse gas pollution. Ruminal methane is produced by a specific class of micro-organisms and knowledge on animal-microbe interactions may open new ways to mitigate livestock environmental impact by managing or selecting animals to reduce the population of methanogen bacteria they host.

To unravel biological complexities, genomic data will be more and more integrated with data collected by other—omic technologies (transcriptomics, proteomics, metabolomics, epigenomics), still at an early stage of application. For example, the combined analysis of gene variation, expression, regulation by microRNA and other epigenetic signals may explain interactions between genes and the environment. These interactions are important in understanding adaptation in a time of rapid climate change and expansion of endemic diseases out of their traditional area, and to optimize animal management to increase production efficiency and animal welfare. The advantage of integrating different—omics data is clear and better methods of data analysis need to be developed, to be able to manage and combine billions of data produced by different approaches and facilitate their interpretation. Biostatistics and bioinformatics are a real urgent need for the scientific community. Novel models of analysis, open source software, storage and calculus capacity are key issues for the future of research in the field.

The—omic toolkit for research and application was recently enhanced with new powerful technologies. Targeted genetic editing is promising to have the greatest impact. The technology quickly edits alleles into more favourable ones without gene transfer across species or between animals. Therefore, it may impact less than the debated GMO technology on public concern, particularly if the first experimental applications will target traits increasing animal welfare (e.g. major genes relevant to adaptation, disease resistance, etc.). However, further research is needed to prove the surgical precision of the intervention, and regulation and ethical aspects of the use of this powerful technology are to be publicly debated before taking genome editing from the lab to the field.

The wrap-up conclusion was that international collaboration and public availability of data are fundamental for the quick progress of knowledge and its translation in practical applications. Partnership and coordination between public funders, research and industry within and across countries, the avoidance of duplication, the exploitation of synergies, the triggering of cooperation and the improvement of local knowledge are the ways to proceed to improve the efficiency and efficacy of research directed to both global and local problems in livestock breeding.

To share knowledge, opinions and technology updates with a larger scientific community in an “open-access” mode, the most relevant ISAFG2015 lectures have been transformed in a collection of papers published in the journal *Genetics Selection Evolution*. We hope you will enjoy reading them.
