# Erratum to: Commentary on the 6th International Symposium of Animal Functional Genomics

**DOI:** 10.1186/s12711-017-0306-5

**Published:** 2017-02-28

**Authors:** Paolo Ajmone-Marsan, Alessandra Stella

**Affiliations:** 1 Institute of Zootechnics, Università Cattolica del Sacro Cuore, Piacenza, Italy; 2 Nutrigenomics and Proteomics Research Center, Università Cattolica del Sacro Cuore, Piacenza, Italy; 3 National Research Council, Lodi, Italy; 4 Parco Tecnologico Padano, Lodi, Italy

## Erratum to: Genet Sel Evol (2016) 48:97 DOI 10.1186/s12711-016-0276-z

After publication of this paper [1], we noted that the “Acknowledgement” section was incomplete and should be as follows:

**Acknowledgements**

This editorial is part of the collection ‘ISAFG2015’ (6th International Symposium on Animal Functional Genomics, 27–29 July 2015, Piacenza, Italy). The publication of the papers in this collection was partly sponsored by OECD Co-operative Research Programme: Biological Resource Management for Sustainable Agricultural Systems (CRP). The opinions expressed and arguments employed in this editorial are the sole responsibility of the authors and do not necessarily reflect those of the OECD or of the governments of its Member countries.
